# Managing COVID-19 waste

**DOI:** 10.2471/BLT.22.020422

**Published:** 2022-04-01

## Abstract

The pandemic is exposing weaknesses in the way medical products are designed, packaged, used and disposed of. Tatum Anderson reports.

Binish Desai knows a thing or two about recycling. “Growing up in Valsad (Gujarat, India) I was surrounded by paper mills,” he says. “As a by-product of their manufacturing processes, they generated millions of tonnes of this unpleasant grey sludge that was just dumped into landfills.”

But where others saw waste, Desai saw a raw material. With just 1500 rupees (US$ 20) in his pocket, and against the wishes of his family, he left home at the age of 16 to start a business turning the sludge into house bricks. Today, combining the sludge with gum base offcuts produced in the manufacture of chewing gum (a base comprised of resins, humectants, elastomers, emulsifiers, fillers, waxes, antioxidants and softeners) and a patented binder, his company produces an average 200 000 bricks a year that are put to a variety of uses, including the construction of buildings.

And now, he is doing the same thing with coronavirus disease 2019 (COVID-19) waste. “We collect face masks from designated collection points at police stations and schools and get face mask offcuts from manufacturers,” Desai explains. “We then disinfect and shred the masks and combine the material with the binder and paper sludge to make bricks that are 52% face mask. We try to do our part, but this is clearly a huge problem.”

A recent report published by the World Health Organization (WHO) gives some indication of how huge. The *Global analysis of health care waste in the context of COVID-19* estimates that tens of thousands of tonnes of health-care waste are being generated, an estimate based on the approximately 87 000 tonnes of personal protective equipment procured between March 2020 and November 2021 and shipped to support countries’ COVID-19 response needs through a joint United Nations (UN) emergency initiative.

“The UN initiative represents only a small fraction of global procurement, but we think it gives a pretty good sense of the bigger picture,” says Maggie Montgomery, a water, sanitation and hygiene expert at WHO, and one of the report’s authors.

As Montgomery is quick to point out, the analysis does not account for the substantially larger amounts of COVID-19 commodities that have been procured outside the UN system, nor the COVID-19-related waste discarded by the public, including over 140 million test kits, with a potential to generate 2600 tonnes of non-infectious waste (mainly plastic) and 731 000 litres of chemical waste.

“Vaccination alone has generated tremendous quantities of waste,” Montgomery explains, noting that the 8 billion doses of vaccine that had been administered globally by 7 December 2021 produced an estimated 143 tonnes of additional waste, including 87 tonnes of glass vials, 48 tonnes of syringes plus needles and 8 tonnes of safety boxes.

“Vaccination alone has generated tremendous quantities of waste.”

Margaret Montgomery

According to India’s Central Pollution Control Board, the country generated approximately 101 tonnes of COVID-19-related health-care waste per day during the first wave of the pandemic in 2020 – adding to the 609 tonnes of waste generated daily by health services there.

Most of that waste was dumped. “Around 70% of India’s city waste is put in landfill, usually on top of a geomembrane that is supposed to protect the groundwater and underlying soil,” says Bhargavi Kulkarni, a researcher at the Rashtreeya Vidyalaya College of Engineering in Bangalore. “Nobody knows how long the geomembrane will remain effective, but there is clearly a serious risk of soil and groundwater contamination.”

Much of the world’s COVID-19 waste does not even make it to landfills. According to a study published in the November 2021 issue of the *Proceedings of the National Academy of Sciences*, 8 million tons of pandemic-associated plastic waste had been generated by 193 countries as of August 2021, more than 25 000 tons entering oceans worldwide.

And then there is the waste that gets burned. “The majority of plastic waste from COVID-19 diagnostic tests is incinerated,” says Montgomery. “Where incineration is not well-controlled, this practice produces toxic pollutants which are harmful to human and environmental health.”

While Montgomery applauds innovative efforts to recycle, such as Binish Desai’s brickmaking project in India, she believes that the key to tackling the COVID-19 waste issue is to generate less waste in the first place.

“Single item packaging is a major concern,” she says. “Initial worries about fomite transmission have led to significant overuse of single packaging and despite abundant evidence that the virus is relatively fragile in the environment, this continues. By not wrapping facemasks and gloves individually, manufacturers could make a huge contribution.”

Overuse of personal protective equipment is another key issue. “I see photos of people vaccinating with gloves on in pretty much every major news outlet. It’s just not necessary,” Montgomery says.

Making personal protective equipment and packaging less toxic and less durable would also help. According to Montgomery personal protective equipment typically contains ortho-phthalates, a group of chemicals used to make plastics, and other substances that are harmful to the environment during the manufacturing process and when products are disposed of by burning.

WHO recommends using less environmentally harmful materials such as nitrile, neoprene and polyurethane in examination gloves rather than polyvinyl chloride and using biobased raw materials in making medical textiles. Examples include compressed hemp, bagasse, polylactic acid and cellulosic fibres as alternatives to plastic in masks, gowns and drapes, and corn-starch-based foam to replace the polystyrene used in vaccine coolers.

Better segregation of waste is also needed. “The problem is that many waste disposal facilities are treating all waste from COVID-19 wards as if it were infectious, whereas in fact it is only materials that have been exposed to bodily fluids that should be considered as such,” Montgomery says. “By lumping everything together, people are often compounding the waste problem.”

Many hospitals have been implementing waste segregation for some time. For example, in King George’s Medical University (KGMU), a 4000-bed hospital in Lucknow, India, colour-coded waste bags and bins are used. Each bin is collected and taken to a centralized receptacle of the corresponding colour, except for the waste in yellow infectious waste bags, which is taken to an incinerator located away from the hospital.

According to Professor Kirti Srivastava, a radiation oncologist, and head of the environment department which oversees biomedical waste management and pest control at KGMU, 90% of the hospital’s waste is non-infectious.

“It was very difficult for treatment facilities to deal with the increased volume.”

Kirti Srivastava

“Black bag general waste is taken away to landfill by the municipal authorities. Blue bag waste (metal and glass base waste), white (sharps) and red (tubing, bottles, catheters and urine bags) are wet-sterilized using steam at 121 °C in dedicated autoclaves on site,” Srivastava explains. Suitable autoclaved disinfected waste is shredded and sold to certified recyclers, generating revenues for the hospital.

Though robust, the system faltered during the first wave of the pandemic when, as in many other countries, national guidance erred on the side of caution and recommended treating all COVID-19 waste as infectious. This led to a sharp increase in yellow bag waste. “It was very difficult for treatment facilities to deal with the increased volume,” Srivasta says. Fortunately, the increase was offset by a decline in waste from non-COVID-19 patients, few of whom came to the hospital during lockdown.

On the incineration front, WHO recommends incrementally improving and replacing burning with cleaner technologies. Options include autoclaving and dual chamber incineration with flu gas treatment. These technologies produce less toxins but are expensive. Lacking resources, many hospitals continue to burn waste in the open or in inefficient, older incinerators.

In some countries, the affordability argument is being challenged. “Researchers compared using waste companies to take infectious waste to low-temperature incinerators on the one hand and autoclaving and then treating the output as general waste for landfill on the other,” explains Dr Oyuntogos Lkhasuren, WHO technical officer in environmental health in Lao People’s Democratic Republic.

They found that the incinerators did not always achieve full combustion and produced carcinogenic emissions like dioxins and furans. They also found that, despite the upfront investment needed to switch to autoclaving, there would be cost-savings over the long term because autoclaved waste could be handled at a lower rate by local waste disposal companies. According to Lkhasuren, the government has taken the bold step of phasing out incinerators altogether, investing in 70 autoclaves to sterilize infectious and sharp waste.

For WHO’s Montgomery, while the pandemic may have exposed a number of issues in the way we generate and dispose of waste, it is also providing valuable lessons in what can be done to address them. “Innovations in PPE (personal protective equipment) design, optimized packaging and delivery, and smart segregation and disposal of waste indicate that we are not faced with an either–or choice between addressing COVID-19 and preserving the environment,” she says. “Learning lessons of the pandemic will help ensure that responses to future health emergencies will promote and protect human and environmental health – leading to safer and more resilient health systems and communities.”

**Figure:**
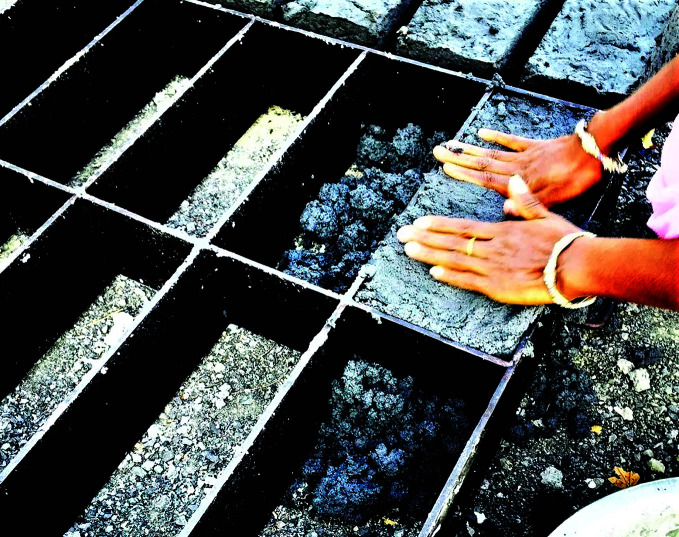
Recycling COVID-19 masks in the manufacture of bricks.

Courtesy of Binish Desai

**Figure:**
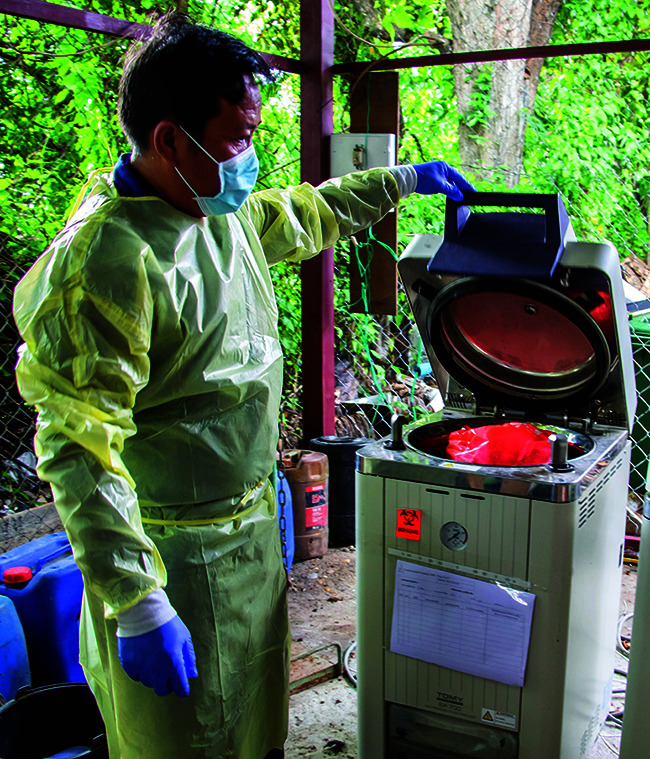
A health worker autoclaves infectious COVID-19 waste in Lao People’s Democratic Republic.

WHO/Vannaseng Insal

